# The Medical Profession of Scotland, and the Lunacy Act of That Kingdom

**Published:** 1861-07

**Authors:** 


### THE MEDICAL PROFESSION OF SCOTLAND, AND THE LUNACY ACT OF THAT KINGDOM.

On Friday, the 7th ult., an important meeting of the medical profession of Scotland was held in the Hall of the Royal College of Physicians, for the purpose of considering the propriety of obtaining some modification of the provisions of the law now affecting the insane. Among those present were:—Dr. Alexander Wood, F.R.C.P.; Dr. Douglas Maclagan, P.R.C.S.; Drs. Andrew Wood, Newbigging, Combe, Halliday Douglas, Seller, Maclagan, Peddie, Hamilton, Coldstream, Andrew Thomson, Keillor, John Brown, Charles Bell, J. Gairdner, Zeigler, W. T. Gairdner, John Struthers, Charles Wilson, Huie, Fairbairn, Wright, Scoresby-Jackson, Burt, Brodie, Jamieson, Myrtle, Malcolm, Murray-Thomson, Johnston, Sibbald, Benjamin Burt, Omond, H. Johnston, W. Brown, Cruickshank, Pridie, R. P. Ritchie, J. Coxe, T. Balfour, Stewart, Burn, and Somerville; Messrs. B. Bell, Carmichael, and A. Dickson; Dr. Smith, Lasswade; Dr. Sanderson, Musselburgh; Dr. Brown, Melrose; Mr. Mackenzie, Kelso; Dr. Brodie, Morningside; Dr. Craig, Ratho; Dr. Hislop, East Linton, &c. &c.

On the motion of Dr. Newbigging, Dr. Alexander Wood, in the absence of Professor Christison from indisposition, was called to the chair.

Dr. Alexander Wood, after expressing his regret for the absence of Dr. Christison and other gentlemen, who were prevented from being present, said that he believed the object of this meeting was pre-eminently one for the good of the public, and more especially of that unfortunate class of the public who required medical treatment as persons more or less deprived of reason. There were two points of view in which they could look at a question like this. One was the point of view which the public were too apt to take, the other the point of view which the medical profession necessarily took, and which he trusted in this meeting would receive a prominence and attention which it was not likely to receive in a public meeting or from the Legislature. If they looked to the reports of Parliamentary Committees—of Parliamentary Commissions he would speak immediately—if they looked to the various legal writings on insanity—if they took the general impressions of the public, as they could gather them in conversation, or as they could read them either in the imaginative pages of the novelist, or in the dry details of legal authority, they would see at once that there was one idea predominant with the public that the great object of all legislation in regard to lunacy should be this—to protect the patients as much as possible from the chances of being unnecessarily immured in asylums, or of having their liberty tampered with in any way. But they, as medical men, looked at the question from the other side, and regarded it in a very different point of view. They must look upon these unfortunates as afflicted with a disease, perhaps the most serious to which the human frame was subject. They must look upon them as afflicted with a disease which statistics prove indubitably to be one of the most curable acute affections, if taken at an early stage, to which man is subject; and one of the most incurable, if neglected in its early stage; and, therefore, their great object was to protect lunatics from the affliction incident to the disease, and place them in the condition most favourable to their cure. Thus there were two antagonistic principles, which were continually struggling the one against the other in all discussions on the management of lunatics—the idea of the public and of legal functionaries that lunatics required protection against medical treatment, and the natural idea of the doctors that lunatics required medical treatment, and ought to have it as early as possible. There was another peculiar element which came into consideration in the treatment of lunacy. In the first place, no lunatic almost admitted his unfortunate condition; and, in the second place, the treatment of lunacy required a certain amount of seclusion, a certain amount of deprivation of liberty which the law was naturally jealous of allowing, and which he would not wish to see the law less jealous of allowing than it actually was. The great object, then, which the Legislature should have in view in framing regulations, was to afford the utmost protection to the liberty of the subject compatible with the early treatment of the disease. They found from statistics that of the lunatics placed under treatment within the first three months after the attack of the disease, the proportion of recoveries were as four to one ; after the first three months, the proportion fell from nine to one ; and after twelve months, the recoveries were found to be only one in four. So far advanced were some Governments in the knowledge of these facts, and in acting on them, that in the Grand Duchy of Baden gratuitous treatment of lunatics is offered in an excellent asylum to all who apply within the first six months. Speaking, as he did, in a meeting of medical men, he need not urge on them that there was no way of treating the great majority of cases of lunacy at all to be compared to the treatment of it in places appropriate for the purpose. Every writer on insanity had contended for this ; and knowing, as they did, the causes of insanity, and how it was aggravated and kept up, they could easily see that it was essential that the patient should be placed in such a position as to be free from all interference with his comfort, or from the despotism of his own temper and disposition, and placed in the condition most favourable for cure. As they all knew, in insanity both body and mind were affected ; and after rude medicine had done its part in removing the complaint of the body, it remained for them by moral treatment to do what they could for the restoration of the mind. It was a singular fact, as illustrating the difference between lawyers and medical men on the subject, that while a few years ago a royal commission was charged with the investigation of the subject of lunacy in Scotland—and charged, as he believed, to make out a case for legislative interference—they could not, on their own responsibility and knowledge, point to any one case of an individual being confined in an asylum whose health did not absolutely require that confinement, but they did point to hundreds of cases where individuals, from the legal impediments thrown in the way, were not confined in asylums who ought to have been confined. They pointed to the case of lunatics undergoing a life of wretchedness, misery, and domestic restraint, under the worst possible conditions for cure ; and while they could show very little of improper treatment of lunatics in asylums, they could show a great deal of very improper treatment of lunatics in their own houses or in the wretched places to which their families had consigned them. What was the fact? In every county in Scotland where lunatic asylums of any note existed, and where there were facilities for removing patients to them, very few were left to be treated in their own homes, but where they were at a distance from asylums, where facilities were therefore diminished, these unfortunates were left to the domestic management, and the aggravation of their disease was the consequence. The necessary inference was that the more they multiplied impediments in the way of removal to an asylum, the more they increased the chronicity of the disease of the wretched patients, and multiplied the very evils it was their object and intention to cure. Another striking fact which no one could read the evidence of the Scotch Commission without noticing was this, that in referring to the duties devolving under the old Act on the College of Physicians, when they had to receive returns from the sheriffs of counties in regard to lunatics confined in these counties, and had to nominate three medical men from whom the sheriff chose a medical visitor who accompanied him round the asylums—while no one could read the report of that Commission without seeing that there was most ample evidence that the medical men did their duty to the best of their ability, as indeed the minutes of the College of Physicians showed, for they had to complain again and again of the irregularity of the returns—there was the most ample evidence to show that the sheriffs did not license houses at all proper for the reception of lunatics, but sometimes licensed houses notoriously improper ; and in one of the most important counties in Scotland the Commissioners found that any man who chose to pay for the licence would receive it, whether his house was fit for the reception of lunatics or not. He argued from this that the legal treatment of insanity was not always the most perfect ; and as to the medical men, they had, he thought, shown that they were willing to do, and had always done, their duty in all respects as they ought to have done in this matter. Their object was to discover how they could best remove the present restraints and interference with the proper and early treatment of lunatics, without at the same time trenching on the liberty of the subject, or giving cause for persons to suspect that it was within the limits of possibility that they or their relatives should be confined unnecessarily in an asylum. He would say this, that the more they created suspicions that the highly-educated and accomplished medical men who opened asylums for the reception of lunatics were persons capable of turning this into a matter of trade ; or persons capable of all the enormities that novelists had pictured, and legalists had imagined in regard to lunatics—the more they created suspicion by unnecessary legal forms and impediments, the more they increased the unwillingness of patients to go into asylums, and of friends to allow them to go. Here, then, were their propositions—asylums were necessary for the cure of lunacy ; early removal to these asylums gave the greatest possible chance of recovery ; deferred removals increased the tendency to chronicity of the disease, besides greatly aggravating the sufferings of the patient. The problem they had to solve was, how were these objects to be accomplished without throwing too loose the regulations, and allowing persons to be received or improperly detained in asylums who ought to be at large? Dr. Wood concluded by saying that, as difference of opinion might exist as to some of the questions to be brought forward, the committee who had prepared the resolutions to be moved, desired that the utmost freedom of discussion should be given, and that any amendment on the resolutions that might be moved should receive full consideration.

Dr. Douglas Maclagan rose to move the first resolution, which was as follows :—"That the present medical certificate is unsuitable. That it should simply bear that the undersigned medical men separately visited and examined (A. B. on such a date), and found him to be of unsound mind, and requiring confinement in an asylum. That this certificate, coming from two qualified practitioners, appears to be amply sufficient, and to require no statement of facts to be appended to it." In supporting the resolution, Dr. Maclagan said he thought there would be very little difference of opinion in the meeting on the propriety of the change proposed in the resolution. Every one who had had occasion to sign these certificates must have found them to be troublesome to fill up—they must have found it most difficult to say what they were to put in and what they were not to put in, and also most difficult to find space in the schedule for writing what they determined to put in. The very size of the schedules showed that those who enacted that these schedules should be used intended that they should be filled up in few words. Now, it was quite possible to describe many diseases in a few words ; but the idea of giving in a line or two—where there was room for only twenty or thirty words—such an account of a case as would convey any information to any human being he held to be a literary impossibility. He had never been able himself to write one of these certificates, so that if it were put before himself by any other person, it would have been satisfactory to his own mind. It was, no doubt, satisfactory to him, because he was conversant with the patient, and was convinced that he was certifying what was true ; but that was a different thing from writing a certificate which would be satisfactory to another medical man who was not conversant with the patient. And if the certificate was not satisfactory to medical men, to whom else could it be satisfactory? He did not think it could be satisfactory to the public mind, wherever that psychological entity might reside, and it could not be satisfactory to the authorities, whoever they might be. He did not think it was possible in the space of a certificate of that kind to convey such information as that an individual, having no other information but that which was contained in the certificate, could come to a deliberate opinion upon the nature of the case, or upon the suitability of that case for confinement. And yet that was precisely the position in which they stood ; for that form of certificate was to be put before a public functionary, and he was to judge of that. If he was not to judge of it, it was unnecessary, if he was to judge of it, it was unsatisfactory. It would be much more satisfactory to him if they were compelled to give in a full report of the case rather than the present certificate. They would then be able to give in something which would convey information, whereas at present they conveyed no information at all. The result was that, coming before the public authorities, different views were taken with regard to these cases. Sometimes the Sheriff said—"It is not my province to judge of these facts at all—I have simply to see that I have a certificate that the man is insane, and that it is signed by two respectable medical men, and I give my warrant." Another says—"There are statements of facts before me, and I shall judge of them," and he proceeds to do so ; and he who has never seen the patient, and who probably knows nothing upon the subject, gives a decision either in support of the certificate, which is unnecessary, or adverse to it, which is detrimental to the patient on those unsatisfactory grounds. He thought, therefore, that the present form of certificate was unnecessary, and did no good, and that the old form of certificate, which was proposed in the resolution, was amply sufficient, while, under that form, it was found that on inquiry there were no instances of parties being improperly put into asylums.

Dr. HUIE seconded the resolution, which was unanimously agreed to.

Dr. MACKENZIE, Kelso, moved the second resolution, which was as follows :—"That the period during which a person may be confined on an emergency certificate is too short, and should be extended from twenty-four hours to three days." In supporting the resolution, Dr. Mackenzie said he must first acknowledge, on the part of the country practitioners of Scotland, several of whom he saw present, the courtesy shown them by the gentlemen who had kindly undertaken the preparation of these resolutions, in inviting them to take part in to-day's proceedings. It was not very long since country practitioners had to be content with what was given them by their town cousins ; but they now felt better days dawning on them. As to the resolution, he did not anticipate any difference of opinion upon it, and if the necessity for extending the time granted by an emergency certificate from twenty-four hours to three days was acknowledged by urban practitioners, it *à fortiori* was necessary for patients residing at great distances from asylums. Dr. Mackenzie spoke of the exhaustion experienced by insane patients by travelling, and the necessity for some days' rest before returning even to the state they were in before leaving home. He referred specially to his own district, stating that they had the average number of insane, and no asylum within thirty miles of them, so that it might be supposed he was earnest in moving this resolution.

Dr. CRAIG, of Ratho, seconded the resolution, which was unanimously adopted.

Dr. PEDDIE said—The resolution which I have been requested to move is the following :—" That many cases of excessive intemperance depend on disease, and constitute a form of insanity. That such cases cannot be treated without confinement more or less strict. That in the present condition of the law, such treatment is frequently unattainable. That some cases of the kind require treatment by confinement, not different from that enforced on other insane persons. That for many more a different system of treatment is desirable. That although such a system of treatment has already been established in various institutions in Scotland, into which persons are admitted with their own consent, yet it seems necessary, in certain cases, to afford the means of enforcing admission into such institutions ; and that such institutions should be licensed, subject to the jurisdiction of the Lunacy Commissioners, and conducted under such regulations as the Act may direct." This resolution is quite a speech in itself for length, and is so explicit and complete in all its parts, that, but for public considerations, in a meeting of medical men such as this very little exposition would be required ; for all here must know the psychological aspects and bearings of what has been called the drinking insanity, termed also, whether correctly or not, dipsomania and oinomania ; and the greater number of us must have had more or less practical and troubled acquaintance with cases of it at various times in the course of our professional experience. I question if any one present entertains a doubt that this phase of excessive intemperance is, what the resolution affirms, a form of insanity—a disease of the brain and mind, however much it may seem in certain instances to spring out of a course of mere moral delinquency, or be mixed up with the vicious feelings and practices of a depraved heart. We have around us abundant examples of the ordinary vice and habit of intemperance, and of the varied ways in which it influences health and conduct. These constitute a monster evil of our country, but they fall within the ordinary limits of transgressions against nature, morality, and society, which imply responsibility and accountability ; and, therefore, the infringements of the laws of health, of social order, common decency, and religion, bring with them their respective penalties in sickness and impaired health, the censures of society, or it may be the degradation of the police barrow and cell, or the punishment of a court of law, or the discipline of the Church. But the condition which is indicated in this resolution lies beyond the boundary line of moral or religious persuasion, or of prosecution for criminal misdemeanour. It is a morbid state of the brain which influences the operations of the mind, chiefly in the way of craving for strong drink, which no secular or sacred considerations can control; and it is developed during a long course of intemperate habits, or exists even at an early age in consequence of strong hereditary predisposition. The transition from the habit or vice into the disease of drinking may be slow in some cases, and in thousands it never takes place at all, although the drinkers may drink often and deep—nay, although in one class of cases they may tipple all day, in another get drunk every night, or in a third, go on a ramble for days or weeks at a time. In a considerable number of instances, however, the vice passes quickly into the disease, and when such is the case we generally find that the individuals affected are of a highly nervous and excitable temperament, with perhaps some family tendency to intemperance. I believe, however, that a large proportion of insane drinkers inherit the tendency from those who were in the same condition as themselves, or were affected with *delirium tremens*, or with mental disorder in some other form. Many of the cases of this malady which I have seen have been so associated. In the very last which fell under my observation, three of the family appear to have been insane, one intemperate and subject to *delirium tremens*, and five highly nervous and hysterical. Dr. Thompson, of the Perth prisons, communicated—in the *Edinburgh Medical Journal* for April, 1858—several most interesting cases of this kind; and I am sure that many of us present could readily multiply such melancholy illustrations. Now, it has been objected, especially by lawyers, who are apt to view the subject in the light of crime, and the clergy, who view through the dark atmosphere of sin, that although there may be no great difficulty as to the propriety of placing the very worst class of insane drinkers under legal restraint for a time, it is almost impossible to draw the line between the lesser degrees of what we doctors call the disease, and the more ordinary forms of the vice of intemperance, and they tell us, if you cannot do so, you might with legal facility, any day, in any of our large towns or cities, place in asylums some hundreds or thousands of drunkards, if there was accommodation for them. Now, I think you will agree with me in replying that we do not find it difficult to discriminate those cases in which legal interference is necessary from those in which it is not, and that all our present difficulties arise only from the want of a clear understanding of the law, and the uncertainty in its administration. In connexion with the one distinguishing feature of the disease, continual craving for, and the excessive use of, intoxicating liquors, there is the other—viz., a total loss of self-respect and self-control in gratifying the desire, and as effects from those, there is the unfitness for performing the ordinary duties, and for fulfilling the obligations of life; also, characterised, perhaps, in one class of cases by wastefulness and extravagance; in another, by indecent or profane conduct; in another by theft; in another by vindictiveness; in another by tendency to impulsive violence, which may lead to the commission of murder or suicide; and in all by extreme deceitfulness and lying. Such afford unmistakable features, when more or less combined, to guide us in our decisions. Then, if we come to review the history of an individual case, perhaps we may, as I have duly noticed, find in it a constitutional predisposition to mental disorder in some form, and whether or not we will learn that ordinary measures, such as moral and religious considerations and friendly restraints, had been employed, but without avail, for the security of person, or property, or family peace and comfort, or for social order. With such facts as these, we need be at no loss how to act, feeling convinced that an insanity is fully established, and that nothing short of personal control, and a humane curative treatment pursued for a considerable period of time under such control will overcome the craving for strong drink, and perhaps effect permanent reclamation. Now, legal facilities for enforcing control in this insanity, which ought to be accorded by a humane public and a wise Government, is in the present state of the land unattainable, except, perhaps, in some of the most extreme cases in which delusions exist, or which are characterised by furiosity; and even in regard to such cases, the uncertainty in the administration of the law is now so great, and the chances of sufficiently long detention are so small, that medical men are becoming more and more unwilling to certify at all. Indeed, in some counties, there appears to be no chance of obtaining the Sheriff's consent, however urgent the case may be, even although there is threatened suicide or danger to the life of others alleged, should anything in the filling up of the schedule, which requires facts, be said about the unsoundness of mind being produced or distinguished by excessive intemperance, or uncontrollable craving for intoxicating liquors. This is surely either a high degree of legal subtlety and mystification, or a very intensely morbid sympathy with the intemperate. Be this as it may, an incalculable injury is thus perpetrated on the unfortunate victims and slaves of a demon propensity, under the abused name of liberty, and a gross injustice done to the sufferers connected with them, and to society at large, whose liberties and privileges ought rather to be protected than thus exposed to infringement and disturbance. When such are the obstacles in obtaining and sufficiently prolonging legal control over the worst class of insane drinkers, how much more difficult it must be to accomplish it, and attempt to effect a cure in those which though comparatively mild, are yet instances of the disease so serious in their effects, that the victims, unrestrained, go on pursuing a ruinous course for time and eternity, and ruinous also as regards others dependent on or connected with them, and subversive of the peace and order of society. Many painful illustrations laying open deep griefs might be given, showing the various ways in which families have been distressed, disgraced, or made destitute by the uncontrolled and uncontrollable course pursued by dipsomaniacs of both sexes. But enough has been said on this point, and I think it will be admitted by all here having experience of such cases that their acts are attributable to a diseased condition of the brain and mind, and not simply to moral depravity, and consequently not to be treated simply by the rebukes or by the discipline of Church Sessions, Presbyteries, or Assemblies. Nothing will do short of such personal control as removes the opportunities to obtain intoxicating liquors; as affords the means, and the time of nourishing and invigorating the body, and of improving the mind—more especially in the exercise of self-respect and self-control, and in leading it into healthier channels of thought and feeling. I will venture to say that had the young minister of Newhills—whose case has been so much before the public of late—been taken charge of by his friends as a dipsomaniac, months even before he was pitched out of his gig and received injuries, which must of course have made his mental condition worse, and more especially if he had been suitably cared for after that accident, he might have been checked in pursuing the mad course of debauchery in which he was hurried along, and instead of being, as he now is, of really, perhaps confirmed, unsound mind, under Dr. Skae's care, he might possibly have had some chance of future usefulness in the Church and in the world. But whether that might have been the result or not, the reasonings of some members of the Aberdeen Presbytery on the whole case show the need of greater public enlightenment on the condition of insane drinkers. It is no doubt praiseworthy for ministers of the gospel unhesitatingly to tear off the cloak designedly thrown over moral responsibility, and give cautious credence to subtle materialistic modes of accounting for the sins of men on the ground of physical and mental conformation; but the views of medical men of experience and respectability ought to be regarded with due respect on matters in which they should be the best judges, when they accord with all that has been written and said of late on the subject of insane drinking. Medical opinion has come boldly and decidedly out of late on this subject, and the public mind—if I may judge from the press—runs more strongly in support of medical views than might probably have been anticipated where there is so much jealousy naturally felt for anything which threatens encroachment on the liberty of the subject. Let us hope that the heavy piece of ordnance recently fired off by our much respected and distinguished medical chief, whose absence we much regret both for the cause of it and the might of his name and character, together with the influence of this meeting, may hasten the humane and beneficial arrangements which are now proposed. Antiquated legal notions and technicalities may somewhat retard the progress of the movement, but I feel confident that the legal mind generally has undergone great change on this subject within the last few years, and from what has been written and stated to myself by men exercising important judicial functions in the country, there appears to be every wish that something should be done in the matter; and I believe that most of the sternness and opposition with which the subject has been met in some other quarters, is not so much owing to the want of enlightened views as to the nature of the drinking insanity, as the necessity imposed on them to administer the law as it at present stands. It seems, therefore, to me most desirable that the law be changed recognising that form of insanity which is produced or manifested by excessive habits of intemperance, and that with any guarantee for the protection of the sacred rights of British subjects, so that its restraints may not be viewed in the light of punishment and mere personal control, but in the humane spirit of securing for the insane drinker a greater amount of immediate happiness, and the prospect of that arising from ultimate reclamation. There is just one topic which I shall briefly notice before closing, and that is referred to in the last sentence of my resolution. At one time I was of opinion that enactments distinct from any Lunacy Act should be pled for, from the idea that such an arrangement would be more agreeable to the popular feeling of the country; and that refuges and sanitaria specially devoted to the treatment of this class of the insane, distinct from other lunatics, might spring up in various parts of the country. But I see the difficulty of recognising any as insane persons without including them in an Act for the insane; and it may be quite possible to obtain a few separate clauses in a general Act recognising the existence of such institutions, while it makes suitable arrangements for their supervision and regulation. Then, too, by a general Act, ordinary asylums may be more conveniently taken advantage of for the cure of insane drinkers, in those districts of the country where such refuges or sanitaria for the poorer class of patients do not exist, and also that compulsory detention of the dipsomania class among paupers in workhouses may be properly legalised, which is not the case at present. The Lunacy Commissioners, in their last report, have drawn attention to this subject, in reference to those inebriates compulsorily detained in the Edinburgh House of Refuge, and they very properly ask the official supervision of such establishments. This is highly proper, and would rather be an encouragement to them than otherwise. With the arrangements of the inebriate department of the House of Refuge, and the good done by them, and their difficulties for want of legal powers of admission and detention, perhaps our excellent friend, Dr. Fairbairn, will kindly inform us. I am only acquainted with four other institutions in Scotland for the cure of dipsomaniacs, but they are all on a small scale, receiving only a very limited number of boarders, and necessarily, in order to be remunerative, those of the highest class. I believe if law took that cognisance of insane drinkers which we desire by this resolution, establishments solely for their treatment would spring up, suitable for all classes, and be more than self-supporting. The amount of good would be incalculable which these institutions might accomplish, by affording occupation, and amusement, and instruction, and promoting a gradual re-introduction to society, and the discharge of civil privileges. In regard to the private establishments at present existing—two in the Island of Skye, one at St. Catherine's, Lochfine, and one near Clackmannan—I have only had an opportunity of visiting the latter—viz., Mr. Sword's, at Kennetpans, and have found that his establishment accommodates only twelve boarders, but that it is excellently conducted, just on the principle of a well-regulated family. The difficulties acknowledged in the conducting of these private establishments are those which might naturally be expected—viz., no power on the side of relatives or friends to enforce admission, and no power for the superintendents of these institutions to detain the patients sufficiently long to accomplish reclamation. Thus, Mr. Sword, of Kennetpans, informed me that while, during his experience of seven years, some excellent successes had been accomplished, several boarders had left before any real benefit was done, and some he has had to turn away from his want of power to sufficiently control. Some of these boarders, although going into such an establishment voluntarily, under the pressure of friends, did so with the deceitfulness peculiar to their malady, and some became rebels. Nothing but special enactments can meet these evils and difficulties connected with this important subject. I could have wished to have said much more on this subject, but I have already detained you too long, and now sit down, trusting that there will be perfect unanimity in the reception of this resolution.

Dr. W. T. Gairdner said he had been asked to second this motion, and in doing so he experienced a difficulty which Dr. Peddie must have felt, and which every medical man must feel, in connexion with the numerous—of the unfortunately too numerous illustrations which arise to his mind in speaking of a subject like this, and to which he was debarred from alluding, because there was no class of cases where the medical vow of secrecy must be more strictly maintained than the class now under discussion ; and anything like a hint regarding any particular case which might give a key to it was absolutely forbidden. But one could, he thought, speak to the motion upon general principles to some extent, although he thought there was great force in the remark made by Dr. Christison in that admirable lecture upon this subject which had lately been republished, that whatever abstract and theoretical difficulties occurred when they were thinking over the subject in the closet, he never found any person whether belonging to the legal or any other profession, who, when brought face to face with the facts of an individual case, had very much difficulty as to how it should be dealt with—who when he observed the wide-spread disaster which was caused by this particular form of insanity, did not yield up his scruples immediately on being informed in detail of these facts. It is peculiarly a case where the practical resolves the theoretical—where they might muddle their brains with metaphysics and with law, and they found, when they were brought face to face with the palpable and the terrible facts of individual cases, their doubts speedily removed. Trying, then, to deal with the matter on general principles, and to keep in view the reasonable difficulties that might be felt either by lawyers, theologians, moralists, or physicians—because it was the common ground of all the four—he would remark that there were three very obviously different kinds of cases in which intemperance and insanity might be connected with each other. In the first place, the habit of intemperance might be superimposed upon a previously existing insanity. A person notoriously insane might, like any other person, become addicted to drink ; and if he was at large, he might thereby aggravate his insanity to an enormous extent—aggravate, at all events, its serious consequences to an enormous extent. Or, on the other hand, beginning with intemperance and nothing else, this habit might so mix itself up with the mental conditions of the individual—might so encroach upon his powers of self-control—might so eat in upon all that constituted health in his mind and health in his body—that in the end he became a hopelessly paralysed individual—a hopelessly mentally destroyed individual—a hopelessly insane individual. Or, again, there is that class of cases—and that, he believed, was the class which was not only the most common, but that which led to all the difficulties and doubts—there was a class of cases where the two causes of disease were, as it were, inextricably mixed up—where the disease began gradually—the habit of intemperance began gradually, and the insanity upon which it was founded began gradually, and the two acted and re-acted upon each other, producing an exceedingly complex series of effects, to which, when the mind was applied at some distance from the beginning, it was almost impossible to find any clear and unquestionable metaphysical solution. That was, he believed, the class of cases which gave the greatest amount of doubt. There was even at the beginning something wrong—an eccentricity—it might not amount to anything that would constitute a good claim on the part of the individual to be placed in an asylum—it might not amount, *per se*, to a claim either for protection to himself or for protection to others from his dealings. But it was a peculiarity manifested by various circumstances—not only by the desire for drink, but by various other disorders—by various bad habits or eccentricities of conduct—by various things, all of which showed that the controlling power of the will was seriously diminished, and that the intellectual functions were not working clear and smooth either ; and in the midst of this he takes to the habit of drink, which causes such a fatal aggravation of his other symptoms that what was originally a cause of eccentricity degenerates in a short time into clear insanity, which might be manifested in a form approaching to idiocy, or in any of the various forms of mental disorder—sometimes it was hypochondria, sometimes monomania, and sometimes suicidal tendencies. And other forms of vices besides drinking sometimes intermix with cases of mania and monomania, the forms being exceedingly varied, and the sequences of events being exceedingly varied. This class of cases, every one must admit, were exceedingly puzzling if they were to try to demonstrate the metaphysics of the thing—if they were to try to demonstrate the starting point of the malady, or the exact sequence of the phenomena. Now, in all this class of cases there were legal questions involved, and there were also moral and theological questions involved, and the lawyer and the divine each naturally viewed these questions from his own point of view, and both perhaps with a slight tendency to exaggerate what was their function in reference to each of them. The lawyer was more apt than another man to view the subject on the side of crime, and the theologian was more apt than another man to view it on the side of sin. He did not mean to say that the divine or the lawyer was wrong in his way of viewing it, or that the medical man was right exclusively in his way of viewing it. But he would say that there was something essentially different in the medical man's point of view from the lawyer's and theologian's; and what he wished to point out was that the medical man's way of viewing it was not inconsistent with the others. It was a parallel decision, but not a decision which precluded others from forming their own decision upon their own grounds. The way in which a medical man viewed this subject when consulted simply as a medical man, apart from his going to a court to give evidence, was simply as a practical question of treatment and cure; he had nothing whatever to do with the metaphysics, or with the law, or with the theology which might be mixed up with the matter. And there was a very great advantage, as it appeared to him, to the public, and to the medical man himself, and to his patient, in his being placed in such a position that he could put all these things off his mind, and simply ask himself, in regard to this particular case, "Here is a great evil to be met—what is to be done in the interest of the patient, and in the interest of society to avert the inevitable impending ruin, destruction, loss of health, loss of character, loss of moral principle, loss of reputation, and the inevitable complication of evils that he sees to be impending if that person is allowed to go at large?" Now, he did think that, without assuming infallibility for the medical profession, they might fairly say that this was a very clear and distinct point of view, that, if they keep to their own point of view—if they did not mix themselves up before they were asked with the points of view of other professions—they might fairly be left, in the interests of the public, to settle their own questions in their own way as much as possible, without being interfered with by the law. Moreover, it might be remarked that the medical man did not wish to prejudice the legal solutions of the case. He declares such a person to be an insensate drinker, or an insane drinker, or anything you like to call it; and when he did so he put the personal liberty of that person under restraint upon that ground. But the medical man did not wish to say that that person, regarding whom he had made such a declaration, and who had been placed under restraint on account of that declaration, was not to a great extent responsible for his own condition. He did not prejudice the solution of any question which might arise if that man came into a court of justice. If, for instance, clergymen had to deal with the case of one of their number who was accused of drunkenness, the medical man did not, by declaring that man insane, or an insensate drinker, wish to prejudice the question of how far the accused party was responsible for the state that he had been brought into. The medical man did not take that into view at all; he simply declared what was the present condition of the person, and what was required upon consideration of that present condition; he did not go into the history of the past; he did not wish to compromise the legal solution of these questions. He (Dr. Gairdner) thought it was important to keep this in view, because it was apt to be misunderstood. It was a very common idea that when medical men committed a man to an asylum they wished to pronounce him absolved from all sorts of moral and legal responsibility. He thought that was a total mistake. A medical man, when he put an insane person into an asylum, did not wish to prejudice his capability of making a will; and still less in the case of an insane drinker, if, when free from drink, he was in other respects capable of executing a will. He placed this question, then, on the basis that the medical man did not go out of his department, for he had a great practical function to perform towards society—he had a great duty to perform to society and to the insensate or insane drinker, and he ought to be as little hampered in the performance of that duty as possible. He had to answer the question, "What is to be done?" He had to answer the question in the interest of the persons concerned, and with a view to the individual case; and the law ought to try as far as possible not to put into his head, or complicate his action with, those metaphysical and legal subtleties which they knew embarrassed all those questions. The only objection he could see to this was the idea that prevailed that medical men would have too much in their power, and that the liberty of the subject would be compromised. In regard to that objection he would only say this, that there was no reason whatever in this respect for separating the case of the insane drinker from any other form of insanity whatever. Every person who had to deal with insanity would tell them that there were numbers of insane persons going about in society, in regard to whom it was a very delicate and difficult question whether they required confinement or not. That question must in the nature of the case be left to the medical man. It was a question of treatment—there was no other way of solving the question except by leaving it to those who had the charge of the patient. The law accordingly did so. The law placed certain securities against abuse of their functions, and the medical men acted under these securities, and with those responsibilities to the law. And there seemed to be no good reason whatever why, in the cases of insane drunkards, medical men should be placed in any different position. If they acted in *mala fide*, or from mere carelessness exceeded their duty in this respect—if they were to go on confining all sorts of persons because they had been overcome with drink probably once or twice, or half-a-dozen of times—if they were to go and convert their power in this respect into an engine of oppression—the securities of the law would at once come to bear upon them in these cases just as in other cases. They would be subject to actions of damages and all sorts of difficulties; the Lunacy Commissioners would come in and liberate the persons whom the medical men had put into an asylum, and the medical men would gain discredit by that. And surely the character of a medical man was a sufficiently sensitive plant to make him not like to come in contact with that sort of difficulty. He maintained that if the interests of the public could be trusted in the hands of the medical profession in any other kind of insanity, there seemed to be no reason why the medical profession could not also be trusted with this class of cases also.

The resolution was agreed to unanimously.

Dr. Andrew Wood moved the adoption of the next resolution, which was as follows:—"That it is desirable that the consent of some public functionary be interposed—as is now the practice in Scotland—between the medical certificate and the confinement of the insane person; and that no public functionaries appear to be better suited for this purpose than the Sheriffs of counties and their substitutes." In supporting the resolution, Dr. Andrew Wood said he had, after carefully considering the question, come to the conclusion—and he hoped the meeting would come to the same conclusion—that it was of great importance that the public functionary that was to be interposed—and there must be some public functionary interposed, that was quite evident—between the medical certificate and the confinement of the patient, ought to be those excellent public officers which Scotland enjoyed, while England was so far, debarred from them—he meant the Sheriffs of counties. So far as he had been able to ascertain, the objections which had been brought against the Sheriff's jurisdiction were chiefly these—first, that in consequence of the necessity of obtaining a warrant from the Sheriff before a patient could be confined there was, in the majority of cases, a very considerable loss of time, causing injury to the patient by delaying the treatment necessary in his case, and placing the friends of the patient sometimes in dangerous circumstances in consequence of the symptoms developed in the interval before confinement. Now, no doubt the necessary application for a warrant often caused loss of time and trouble—and he recollected of a case where a patient became ill on a Sunday, and had to be kept by his friends till the following day, to his own great danger as well as to the danger of those around him. He thought, however, that in some degree the evils arising from delay had been removed by the introduction of what was called "certificates of emergency," but which he would rather—as suggested to him by a very experienced person in these matters—call "a professional certificate." And if they succeeded, as proposed in a previous resolution, in inducing the Lord Advocate to put in a clause in his bill extending the period during which a person might be confined on an emergency or provisional certificate from twenty-four hours to three days, the objection he was now considering to the jurisdiction of the Sheriff would be got rid of. Another objection might easily be raised to the present system, and it was this. By the present form of certificates, they were obliged to state the circumstances which they had observed in regard to the patient, which led them to consider him of unsound mind. These certificates were sent to the Sheriff, who was thus made to sit as judge over the certificates—that was to say, that while the medical man was held responsible for his certificate, the Sheriff really decided the question of unsoundness. So far as he had seen, the present form of certificate was opposed completely, in his opinion, to common sense, and in practice it had been found by medical men to be most irksome, and he might almost say impracticable. But if the improvement in the certificate suggested in the first resolution should be introduced into a bill, and made the law of the land, another objection to the Sheriff's jurisdiction would be removed, as all he would have to do would simply be to satisfy his mind that the requirements of the Act had been carried out, and that the certificate had been signed by men who, being on the medical register, were properly qualified to sign such certificates. The third, and he believed the great objection to the Sheriff's jurisdiction was this, that it was found that patients and their friends had a great objection to having the consignment to a lunatic asylum put on the footing of a warrant to the Sheriff, in a manner somewhat analogous to the consignment of criminals to gaol. It was argued that this feeling was so strong in the minds of many persons connected with insane people, that the admission of patients to asylums was greatly delayed, and in some cases not carried out at all, greatly to the injury of the patients themselves. He had no doubt that this feeling operated to a very considerable extent, but he would ask, "Are there not a very great many other things that operate in deterring people from sending their friends into asylums?" Was it not a very natural thing that people would not like their friends to go into an asylum at all, because that might damage their prospects in life? This feeling of aversion to sending friends into asylums would therefore continue, whether they required to go to a Sheriff or not; and he thought they must not, therefore, view this question in a one-sided manner. He would be extremely glad to adopt anything which would allay the feeling of aversion to sending patients to asylums, and remove the difficulties at present felt in getting patients promptly under treatment in asylums. At the same time, it must be recollected that the subject must be considered from other points of view. What was it that was proposed by those who wished to do away with the Sheriff's jurisdiction in this matter? It was simply this, that in the case of a British subject they were to be entitled to suspend on this particular case the Habeas Corpus Act. Now, he would say that whatever opinion the medical profession might have in regard to the expediency of having facilities for putting lunatics in asylums, they must have regard to the feelings—or, if they would, to the prejudices of the day, and to the genius of the British Constitution—and in whatever they did they must recollect that there was, and must always be, a great jealousy of any individuals in this country not only being deprived of their liberty, but being deprived of their liberty under circumstances which did not come under the cognisance of some authority whose duty it was to see that there were real and true grounds for the confinement, and that it was not made under improper circumstances. Another objection was brought to the Sheriff's warrant, to the effect that, after all, it was of no use. He was inclined to dissent from that view of the question, because it appeared to him that he should not like to sign a certificate which was to consign any person to a lunatic asylum without a perfect consciousness in his own mind that the certificate would not or could not in any way be abused. And therefore he felt that the fact that the certificate must go before the Sheriff, and be passed through his hands, and be countersigned—if he might so say—by his warrant, that gave him a security that that certificate would not be abused for improper purposes. It might be said that the Lunacy Commissioners would afford the required guarantee against abuse. He would be very well content with the Lunacy Commissioners, but they wanted the element of ubiquity. It was impossible that they could be present at the same time in Edinburgh and in Caithness. That was the advantage of the Sheriff's jurisdiction—that in every quarter of the country they had these most admirable officers situated—men accustomed to administer the law, and who had the Lunacy Act before them, and who, knowing the circumstances, were in a position to know whether all the requirements of the Act had been complied with, and whether the individuals who signed the certificates were properly qualified to do so. If they were to have this power granted which was now asked in regard to insane drinkers, as he trusted they might have—and he felt the importance of the present meeting, if for no other purpose than for the enlightenment of public opinion which the speeches of Dr. Peddie and Dr. Gairdner had given them that day, and he was quite sure that, coming as these speeches did so immediately after those interesting cases which had occurred in their Church Courts, they would do an immense deal to educate public opinion upon a matter which they very little understood at present; but what he wished to say at present was this, that if they were to get this additional power in regard to the confinement of drunkards, he thought the public would look with enormous suspicion upon them if, whilst they were seeking more power in regard to that important class of patients, they might at the same time appear—for it would be no more than appearance—but if they appeared to be getting rid of the guarantee to the public which the superintendence of the Sheriff seemed to give, that that very great power so committed to medical men, and in a certain degree to the friends of the patients, should not be abused. While, therefore, this question of the Sheriff's jurisdiction was an open question, he thought it would be extremely inadvisable—to say the least—to go to the Government or to the Lord Advocate to ask for such a reform—nay, a revolution—in what had so long existed in this country, and to get a change introduced in regard to the doing away with the powers of those Sheriffs whose jurisdiction and administration in this matter seemed to him to be conformable, not only to the liberty of the British constitution, but he would also say conformable to the genius of the people of Scotland.

Dr. John Brown seconded the motion.

Dr. Charles Bell rose and said that, in the absence of Dr. Burt, who had been unexpectedly obliged to leave the meeting, he had been called to move the following amendment to the motion now proposed :—"That it is not desirable that the consent of any public functionary be interposed, as is now the practice in Scotland, and nowhere else required, between the present medical certificate and the confinement of the lunatic; the present system being only derogatory to the profession, being prejudicial to the patient, forming, as has been found, no protection whatever against vexatious prosecutions, and converting that which ought to be a strictly private professional duty into a public judicial act which unavoidably associates it with the idea of criminality. That, as a substitute for the present form of procedure, it should be required that intimation be made without delay to the Commissioners in Lunacy, of the confinement of the lunatic, with copies of the documents on which the removal has proceeded, they being invested with full powers." He (Dr. Bell) thought the amendment he had now read contained sufficient argument in favour of their seeking for the abolition of the sheriff's jurisdiction in the matter altogether. He was not aware of any advantage which had been derived from the sheriff's warrant, and much disadvantage had resulted therefrom, both to the patients, to their friends, and to the medical profession in general. It led to unnecessary delay, and to the undue publication of what should be considered a private matter altogether; while it gave no protection to the medical man against prosecution; and did not add in the slightest degree to the confidence of the relations of patients in sending their friends for proper treatment to an asylum. It had been stated that there had been few prosecutions of medical men in Scotland, and that the small number of such prosecutions was owing to the interposition of the sheriff. He totally disagreed with that statement. The small number of prosecutions in Scotland was, he maintained, to be attributed to the high character of the medical men who had given the certificates, and the upright and honourable conduct of those men who had the charge of lunatic asylums, and not at all to the sheriff's jurisdiction. The necessity for going to the sheriff for a warrant induced people to believe that lunacy was a crime, not a disease. He saw no more reason why they should go to the sheriff for a warrant to send a person to a proper place of protection than there was for their asking authority from the sheriff in prescribing for any serious illness.

Dr. Fairbairn seconded the amendment.

Dr. Sanderson, Musselburgh, said he could fully corroborate all that had been said as to the trouble caused to medical men with regard to certificates that came before the sheriff, the certificates being in many cases treated in a very capricious manner. Dr. Sanderson proceeded to state that a private lunatic asylum having been recently given up in his district in consequence of the death of the superintendent, the lunatics, about ten in number, had to be conveyed to other asylums; and he, as medical attendant of the asylum, gave the requisite certificates, some of which were not received by the sheriff because the patients were to be conveyed to another asylum of which he had the charge, and in which he was supposed to have a money interest. He had no money interest in the asylum beyond being medical attendant in the asylum, but the refusal of the certificate was grounded upon the 71st clause of the Act.

Dr. Douglas Maclagan said the question raised by Dr. Sanderson was simply this, whether a person who was a medical attendant of any asylum, and who received fees in that capacity, had a pecuniary interest in it, according to the meaning of the Act; and that was precisely a question for a sheriff to determine. Supposing there had been no sheriff, and Dr. Sanderson had put in these patients into that asylum, and they had afterwards come out and raised an action of damages against him, he might perhaps have suffered under that clause. The sheriff had, therefore, not injured Dr. Sanderson, but protected him by his interference. He was not saying that the clause in the Act referred to by Dr. Sanderson was right—it might be absurd and unnecessary—but the question was whether, with such an enactment before them, it was not a better thing for them to have the sheriffs there, and whether there did not occur questions where the interposition of the sheriff was an important thing for them. The fact that there were fewer prosecutions of medical men in Scotland than in England was undoubted; and there were two theories put forward to account for this—the one being the jurisdiction of the sheriffs, and the other the high character of the medical profession. He was not there to say anything to lower the character of the medical profession in Scotland. He thought they were able to stand their own ground in that respect; but he was not so sure that they were entitled to be quite so uplifted upon their own position as thus to cast a comparative slur upon the medical profession in England, and to say that it was because the Scotch doctors are superior men that English doctors were liable to actions of damages, while the medical profession in Scotland got off for their very high character. Then there was another dilemma into which they would be placed by insisting on this theory of the high character of medical men accounting for the small number of prosecutions, and it was this, "Is it the want of high character that has been the cause of the prosecutions in the cases of those medical men in Scotland who have been subjected to prosecutions?" That was a most serious dilemma into which this theory placed them—and if he had been one of those unfortunate individuals against whom accusations had been brought—and he was very nearly one—he should be very sorry if anybody should say of him, "There is one of those unfortunate fellows whose want of high character has subjected them to prosecution." He was of opinion that it was the jurisdiction of the sheriff, which distinguished them from England, that had been their protection, and he thought it would be a great pity to do away with that. And he attached great importance to the argument of Dr. Andrew Wood that, while they were asking an increase of power, it would be a pity if they should also be asking, at the same time, to do away with the only thing that appeared to be a *habeas corpus* protection to this class of patients. He believed that, by adopting the amendment of Dr. Charles Bell, they should do a great deal of harm to the cause of this most important meeting.

The Chairman hoped that some member of the committee who differed from the majority who proposed the motion now before the meeting, would state the grounds of their regret that such a motion had been brought forward. He held strong views upon the subject, but perhaps in his position as chairman he had better put the motion without saying anything.

Dr. John Gairdner asked if it was necessary that the meeting should affirm either the proposition of the motion, or that of the amendment. Would it not be better for them simply to say that, provided what they had suggested in the former resolutions was adopted as the basis of an amended Act of Parliament, they were perfectly willing to subscribe to any plan which, in the wisdom of the Legislature, might seem calculated to secure against erroneous imprisonment, for that, he believed, was the only reason alleged for the interposition of the sheriff.

Dr. W. T. Gairdner said he could not vote personally for either of the two motions. They appeared to him to be both quite too positive on the one side and on the other, and to take up a position which they had not sufficiently argued or thought about. The matter was so complicated with legal technicalities, that he was not prepared to assert that the Sheriffs were the only proper persons to interfere on the one hand ; nor was he prepared to support the amendment. Within the last few days, having in view that there were considerable differences of opinion upon this subject, and being himself uncommitted upon it, he took an opportunity of speaking to two Sheriffs on the subject, and he had these things in his mind when he spoke to them. He thought, "If the Sheriff's warrant is to be of any use as regards the security of the public, it ought to secure persons against the possibility of being confined to an asylum by the certificates of scamps in our profession, and of incompetent persons." He spoke to both these two gentlemen—and both of them were gentlemen who had been considerably criticised in the profession on account of objecting to the terms of the medical certificates, and he said, "Would you consider it part of your duty, if you got a medical certificate signed by two persons, one of whom you privately knew to be a scamp, and the other of whom you knew to be pretty nearly an idiot—would you think it right in these circumstances to ask for more certificates?" They both replied "No." They said, "The law does not ask us to seek more opinions ; the law simply asks us to see whether they are qualified men ; we have no right to act upon our knowledge of the character of these men." I replied, "Well, I don't see the use of you ; you ought to be abolished, and if not, I want you to have better powers for the protection of the public and of the medical profession." He wished the Sheriff's jurisdiction to be either amended or abolished ; he thought that at present it was a mere obstruction. The Sheriff interfered at present where he ought not to interfere ; he interfered with their grounds of confining men, which was no part of his business ; and he did not interfere with what he should interfere with—namely, the question of whether those who signed the certificates were not notoriously incompetent and disabled from doing so. He could not vote either for the motion or the amendment, but he could vote for the following amendment, which he begged to propose :—"That the existing form of the Sheriff's jurisdiction in cases of insanity is objectionable, as tending to delay and obstruction in the admission of cases of urgency into asylums, and also as interfering with the physician's province, which is to judge of the circumstances under which treatment in asylums is required, and that the security of the public requires only that the competency and good faith of the medical men signing the certificate should be placed beyond suspicion. That the Lord Advocate be requested to take these circumstances into his consideration, with a view either to the amendment of the Sheriff's jurisdiction or to its being replaced by some other provision for accomplishing the object in view."

Dr. Ritchie seconded the amendment of Dr. W. T. Gairdner.

The Chairman said he would like to state shortly his views before the vote was taken. The feeling which he had upon the subject was just this—in the first place, the certificate or warrant of the Sheriff was either something or it was nothing. He should assume first that it was something. If it was anything at all, it was a judgment upon evidence laid before him whether the medical men were right in the first instance in saying that the person was insane, and in the second instance in saying that the proper treatment for that person was seclusion. Now, he held that that was a purely medical question, only to be decided by a medical man, and that the interference of a Sheriff in this respect was dominating and ruling over the province of a physician, which he had no right to do in that particular case. Well, then, he should assume, in the second place, that the warrant was a farce—which he believed it was in most cases—he should assume that it was a mere form. He would say, then, that all forms without reality were mere shams and mockeries, and in so important a matter ought to be condemned. If it was a sham, what was the use of it? It increased trouble, created delay, and placed an obstacle between the patient and the early treatment of his disease. Then it invested the asylum with additional horror, for it looks on the one side as a place requiring patients to be protected against it, and on the other as a place of confinement to which patients were sent by a warrant, just as they were sent, by another sort of warrant, to the gaol. It was only the other day that a patient said to him, "Doctor, is it possible that you put me here like a criminal, with a warrant from the Sheriff?" That was a common feeling, and they knew that whilst relations had no objections to the doctors' certificates they continually objected to the Sheriff's warrant, as that gave publicity and a criminal character to the proceedings. In regard to Dr. Maclagan's remarks as to the cause of the smaller proportion of prosecutions in Scotland than in England, he would ask, "Why is it that we have so few prosecutions for malpractice in Scotland? Why do you continually see in England surgeons and accoucheurs being brought up for having treated wrongly fractures and dislocations, and deliveries, while you never hear of such things in Scotland?" When Dr. Maclagan answered that question, then they would probably know that it was not the interposition of the Sheriff that protected the medical profession from these prosecutions in Scotland. He could understand that if the Sheriff sat as a judge in these cases, and if the medical men came before him as witnesses, and if the Sheriff pronounced a judgment, it would be of vast use, for the medical men coming before him as witnesses would be precluded from those unjust prosecutions, just as in civil or criminal cases, when they gave their testimony on oath. But that was not the case; and in the few cases of prosecution that he had been called to see, he believed that the Sheriffs had never been called even to say that they believed the medical men certified what was true. And yet they were told that this was a necessary protection, to see that the forms were observed. If the keepers of asylums, or medical men, did not observe the forms of the Act of Parliament, they acted at their peril, and that was the protection of the patient. As to its being a suspension of the Habeas Corpus Act, why if that was the case, then in England, where no such form as the Sheriff's warrant was required, the Habeas Corpus Act was being violated twenty times every day. The last consideration he would put before the meeting was this, that the great security of the patient, besides the penalties for the violation of forms, was the fact that they were superintended by the Lunacy Commissioners, educated men, and with medical men among them competent to judge of the merits of any case. Although the Lunacy Commissioners were not ubiquitous, as had been said, they went about the country and would very speedily inquire into any case requiring interference; and this ought to be satisfactory to the public. He thought the great danger was in multiplying protections and difficulties instead of giving facilities for the cure of this malady.

Dr. Charles Bell withdrew his amendment in favour of Dr. W. T. Gairdner's, at the suggestion of Dr. Douglas Maclagan, who remarked that some, like himself, could vote for Dr. Gairdner's amendment, though they could not vote for that of Dr. Charles Bell.

A vote was then taken by a show of hands, when Dr. W. T. Gairdner's amendment was carried by a large majority.

Dr. Sellar proposed the adoption of the following motion, remarking that, notwithstanding the little division that had just taken place, they were really unanimous on all the important matters which had been before them:—"That a memorial embodying the opinions of the meeting be presented to the Lord Advocate; that a committee be appointed to prepare this memorial; and that, in the event of his Lordship agreeing to introduce into a Lunacy Amendment Bill provisions in accordance with these general principles, the committee be authorized to confer with his Lordship on the subject."

Dr. Hislop, East-Linton, seconded the motion, which was agreed to unanimously.

On the motion of Dr. Balfour, a committee was appointed to carry out the object of the resolutions.

A vote of thanks was given to the chairman, on the motion of Dr. John Gairdner, which terminated the proceedings.

---

(*Scotsman*, *June* 17.)

At the late meeting of the medical profession on the Lunacy Act, considerable difference of opinion was displayed as to the object and utility of the order which the Sheriff grants for the admission of patients into asylums. When a man becomes insane, and it is considered necessary to place him in confinement, or, in modern phraseology, to detain him under care and treatment, a petition must be presented to the Sheriff for his order, by some person whom the Act has omitted to define, authorizing the reception of the patient into an asylum. This petition must be accompanied by certificates from two medical men that the patient is of unsound mind, and "a proper person to be detained under care and treatment;" and the facts on which this opinion is founded are required to be stated. There are thus three parties implicated in placing a patient in an asylum—the petitioner, the Sheriff, and the medical men. Now, with which of these three lies the onus of the act? With the petitioner? Morally, perhaps, but we should think not legally; for, without the certificates of the medical men that the patient is a proper person to be placed in an asylum, no steps could be taken in the matter; and with such certificates it would be culpable to refrain from doing what the medical advisers recommended for the benefit of their patient. The responsibility must, therefore, lie between the Sheriff and the medical men. The latter, however, are constituted by the Act the only judges of the patient's insanity. They alone are called on to see and examine him, and the duty of the Sheriff appears restricted to seeing that their certificates are framed according to the statutory forms. For aught that the Act says to the contrary, the petitioner may never have seen the patient, and may be resident in a distant part of the country, or even out of Scotland; and the Sheriff, on his part, may be ignorant, not only of the county of the patient's usual residence, but even whether he have a domicile in Scotland. Everything consequently hinges on the medical certificates, and the question accordingly is, whether any check should be exercised over medical men to prevent them granting certificates of insanity without due consideration, and whether, in this event, the Sheriff's order constitutes such a check.

It is a popular belief that the Sheriff's jurisdiction embraces merely his own county. Under the Lunacy Act, however, he is authorized to grant his order for detaining patients in the asylums of *adjoining* counties, and this permission has been used with such latitude that they are sent to asylums in whatever part of Scotland these may be. Accordingly, patients from almost every county will be found in the asylums of Mid-Lothian, admitted on the orders of the Sheriffs of their respective counties. With the existing deficiency of asylum accommodation, it would be difficult for the Sheriffs of many counties to refuse their orders for the transmission of patients to distant asylums; but whether the granting of them is in accordance with the provisions of the statute, is another question, and one which is open to much doubt. But perhaps of even more doubtful legality would be the procedure which the refusal of orders under such circumstances would entail. In this case it would follow that the patients would be removed from their own counties, and carried across various others, to be placed in the asylum of a distant county by the order of a Sheriff, who necessarily would have no knowledge of the patients, and very little, if any, of the medical men granting the certificates. There are in the asylums of Dumfries, Glasgow, and Edinburgh many patients who have been brought not only from distant parts of Scotland, but even from England and Ireland, and who are detained under the orders of the local Sheriffs, granted sometimes on the certificates of practitioners resident within their own jurisdiction, but more frequently perhaps on those of medical men belonging to the places from which the patients were brought. It thus appears that the order of the Sheriff merely guarantees the fact that the statutory forms have been complied with. He knows nothing and inquires nothing about the patient. At one time he sends him a hundred miles or more from home, far beyond his own supervision; and at another he authorizes his detention in an asylum within his own county without troubling himself to inquire whether, if a stranger, he were legally brought within his jurisdiction.

Does the Sheriff's order, then, serve as a protection to the subject? We do not see how it can be regarded in this light. If a patient is brought to Glasgow on the certificates of two doctors of Tipperary, or to Edinburgh on those of two practitioners of Manchester, the Sheriff grants his order without stopping to inquire the character of the men whose names are appended. We do not suppose he even turns up the Medical Register to ascertain whether they are qualified practitioners. He lets those who gave their signatures take the responsibility of the act. In this course we apprehend he is legally right; but then, we would ask, wherein lies the virtue or utility of his order? Dr. Christison regards it as a buffer to protect the medical man from actions of damages on the part of the patient. But even in this respect it is but a sham, a delusion, and a snare, and fails in its purpose. It is possible that the knowledge that their certificates must be submitted to the Sheriff, operates, with some medical men, in rendering them more cautious than they would otherwise be in certifying insanity, and herein, we believe, lies the chief use of the Sheriff's order, and the only valid reason for retaining it. It is to the operation of this cause that we would in some degree ascribe the fact, if fact it be, that the number of actions of damages brought by patients against medical men is less in Scotland than in England. We are, however, by no means sure that, taking into consideration the greater population of England, any such difference really exists. At any rate, we should be glad to see the statistics on which this opinion is founded. And it must also be remembered that in England the number of private asylums is comparatively much greater than in Scotland; and that in the former country private patients are generally placed in these establishments, whereas in Scotland public asylums have the preference. The risk of collusion for the detention of patients may accordingly be supposed to be greater in England than in Scotland, inasmuch as private interests come there more into play, and the fear of having been the victim of such collusion may in a certain degree influence the number of prosecutions. But it has further to be considered that the status of legal practitioners in the two countries may affect the result. It may possibly be more easy to find a pettifogging lawyer in England than in Scotland—that is, one who will undertake the patient's case on grounds evidently frivolous and vexatious. There are, therefore, various grounds for doubting whether Dr. Christison's views may not have been too hastily adopted.

Since the passing of the Lunacy Act the position of the Sheriff in regard to lunatics has been greatly modified. Formerly, he was authorized not only to grant his warrant for the admission of patients into asylums, but he was constituted the statutory visitor of these establishments. Under the new *régime*, the duties of visitation are performed by the Commissioners in Lunacy, and for this reason, among others, we are inclined to think that the authority for the admission of patients, also, should now emanate from them. It is frequently said that, in England, no authority equivalent to the Sheriff's order is necessary for the admission of a patient. In one sense this is true. A private patient is admitted on the order of a relative and two medical certificates; and a pauper on the order of a justice, or that of a clergyman and the relieving officer of the union, and one medical certificate. But in England the order is not a warrant for detention in the meaning attached to the term in Scotland, but rather a permission to extend to the patient the benefits of the institution, and is, in fact, analogous to the order granted by a manager or subscriber for admission into the wards of the Royal

Infirmary. Hence, the order only of a justice of the county in which the asylum is situated, or that of the overseer of a parish within the county, or having otherwise a claim to the admission of its pauper lunatics, would be accepted. The asylum is thus regarded as a benevolent institution, and not as a prison to which patients are committed under a legal warrant. The check against abuse, and one which, we believe, is in reality more efficacious than the Sheriff's order is with us, lies in the transmission of copies of the documents on which admission takes place to the Commissioners in Lunacy. It is their duty to examine these documents, and to judge whether the medical certificates are filled up in accordance with the statute, to call for their amendment when imperfect, and to require the discharge of the patient when the evidence of insanity is unsatisfactory. Under this procedure there occurs no delay in placing the patients under treatment. Accordingly, there is no necessity for certificates of emergency, and the great grievance complained of by medical men in Scotland, of botheration and annoyance from crotchety Sheriffs, is never heard of. It is true that similar documents are transmitted to the Scotch Board of Lunacy, but the Scotch statute does not empower the Commissioners to act on them. The old Scotch and the modern English procedure are both partially introduced into the new Lunacy Act, and, from their incompatibility, are constantly jostling each other. The question, therefore, to be decided is, which should have the preference. We incline to the belief that the English procedure is most in accordance with modern views as to the nature of insanity and the treatment of the insane, and that it is likewise best calculated to prevent improper detention in asylums. At all events, it is clear that some change is urgently called for in a system which allows the patients to fall between two stools, and gives satisfaction neither to the medical profession nor the public.

